# Changes in serum metabolomics in idiopathic pulmonary fibrosis and effect of approved antifibrotic medication

**DOI:** 10.3389/fphar.2022.837680

**Published:** 2022-08-17

**Authors:** Benjamin Seeliger, Alfonso Carleo, Pedro David Wendel-Garcia, Jan Fuge, Ana Montes-Warboys, Sven Schuchardt, Maria Molina-Molina, Antje Prasse

**Affiliations:** ^1^ Department of Respiratory Medicine, Hannover Medical School and Biomedical Research in End-stage and Obstructive Lung Disease (BREATH), German Center for Lung Research (DZL), Hannover, Germany; ^2^ Institute of Intensive Care Medicine, University Hospital Zurich, Zurich, Switzerland; ^3^ ILD Multidisciplinary Unit, Hospital Universitari Bellvitge, IDIBELL, Universitat de Barcelona, Hospitalet de Llobregat, Barcelona, Spain; ^4^ Fraunhofer Institute for Toxicology and Experimental Medicine, Hannover, Germany; ^5^ Centro Investigación Biomédica en Red de Enfermedades Respiratorias, Instituto de Salud Carlos III, Madrid, Spain

**Keywords:** fibrosis, IPF, antifibrotic, metabolome, lipidome

## Abstract

Idiopathic pulmonary fibrosis (IPF) is a progressive disease with significant mortality and morbidity. Approval of antifibrotic therapy has ameliorated disease progression, but therapy response is heterogeneous and to date, adequate biomarkers predicting therapy response are lacking. In recent years metabolomic technology has improved and is broadly applied in cancer research thus enabling its use in other fields. Recently both aberrant metabolic and lipidomic pathways have been described to influence profibrotic responses. We thus aimed to characterize the metabolomic and lipidomic changes between IPF and healthy volunteers (HV) and analyze metabolomic changes following treatment with nintedanib and pirfenidone. We collected serial serum samples from two IPF cohorts from Germany (*n* = 122) and Spain (*n* = 21) and additionally age-matched healthy volunteers (n = 16). Metabolomic analysis of 630 metabolites covering 14 small molecule and 12 different lipid classes was carried out using flow injection analysis tandem mass spectrometry for lipids and liquid chromatography tandem mass spectrometry for small molecules. Levels were correlated with survival and disease severity. We identified 109 deregulated analytes in IPF compared to HV in cohort 1 and 112 deregulated analytes in cohort 2. Metabolites which were up-regulated in both cohorts were mainly triglycerides while the main class of down-regulated metabolites were phosphatidylcholines. Only a minority of de-regulated analytes were small molecules. Triglyceride subclasses were inversely correlated with baseline disease severity (GAP-score) and a clinical compound endpoint of lung function decline or death. No changes in the metabolic profiles were observed following treatment with pirfenidone. Nintedanib treatment induced up-regulation of triglycerides and phosphatidylcholines. Patients in whom an increase in these metabolites was observed showed a trend towards better survival using the 2-years composite endpoint (HR 2.46, *p* = 0.06). In conclusion, we report major changes in metabolites in two independent cohorts testing a large number of patients. Specific lipidic metabolite signatures may serve as biomarkers for disease progression or favorable treatment response to nintedanib.

## 1 Introduction

Idiopathic pulmonary fibrosis (IPF) is a fatal disease with a mean survival time of 3–5 years (Lederer and Martinez, 2018). Two medical compounds have been approved for treatment of IPF. Pirfenidone and nintedanib attenuate the mean decline in forced vital capacity significantly to a similar extent (King et al., 2014; Richeldi et al., 2014; Raghu et al., 2015). However, effect of treatment is not equal across patients. Some patients appear to benefit more, while others rapidly decline despite receiving adequate antifibrotic therapy. While nintedanib acts by inhibition of platelet-derived growth factor (PDGF), fibroblast growth factor (FDG) and vascular endothelial growth factor (VEGF), synergistically leading to downregulation of fibrosis associated pathways (Hostettler et al., 2014; Roach et al., 2021), the distinct mode of action for pirfenidone for inhibition of fibrosis is still insufficiently understood (Conte et al., 2014; Epstein Shochet et al., 2018; Jin et al., 2019; Ruwanpura et al., 2020). There is currently no set of biomarkers available capable of gauging the therapeutic efficacy of nintedanib or pirfenidone (Jee et al., 2019).

The technique of mass spectrometry based metabolome profiling has significantly improved during the last decade with metabolome measurements becoming increasingly robust and reproducible (Yang et al., 2019). The technique was frequently used in cancer research since various cancers induce vast metabolic dysregulation (Schmidt et al., 2021). In contrast to regular cells, proliferating cancer cells have a high demand for energy supply and use glycolysis even in normoxic conditions (Madama et al., 2021). Many therapeutics used in oncology alter cellular metabolism and thereby interfere with cancer cell proliferation. In future, changes in metabolome induced by drugs may serve as biomarker to monitor treatment efficacy (Chung and Griffiths, 2007).

Recently the involvement of aberrant metabolic and lipid pathways have been implicated to affect IPF pathophysiology. Altered metabolism of the amino acids glycine, glutamine and arginine and dysregulated glycolysis was shown to promote profibrotic phenotypes via TGF-ß dependent pathways (Zhao et al., 2017; Gaugg et al., 2019; Roque and Romero, 2021). For lipids, increased levels of long-chain and medium chain fatty acids have been reported in IPF lungs and macrophage reprogramming with increased fatty acid beta oxidation have been described. (Mamazhakypov et al., 2019; Tedesco et al., 2019; Roque and Romero, 2021). Sphingolipids and lysophsphatidic acid (LPA) as other lipids play a part in many pathophysiological processes and were particularly associated with fibrotic processes (Shea and Tager, 2012; Pyne et al., 2013). With most evidence derived from animal studies, smaller analysis involving broad circulating metabolomic and lipidomic profiles from IPF patients showed deregulated profiles (Zhao et al., 2017; Nambiar et al., 2021a; Nambiar et al., 2021b). The impact and correlation of serum metabolomic profiles remains insufficiently investigated.

In the context of these recent findings, we got interested in the metabolome of IPF patients and whether treatment with approved antifibrotic medication is capable of reversing metabolic changes or predicting therapy response. In order to address these questions, we performed serial serum metabolomic assays in IPF patients and healthy volunteers as comparators and correlated disease progression and severity.

## 2 Patients and methods

### 2.1 Patient and sample selection and study design

For this study we retrospectively selected patients who had a confident diagnosis of IPF in accordance with the practice guidelines issued by the American Thoracic Society (ATS) and the European Respiratory Society (ERS) (Raghu et al., 2018) and were started on antifibrotic therapy with either nintedanib or pirfenidone at Hannover Medical School (Germany) as an exploration cohort. A further validation cohort of IPF patients was derived from the Hospital Universitari Bellvitge (Spain). Additionally, age-matched healthy volunteers were screened for pulmonary abnormalities by interview, physical examination and routine laboratory. For IPF patients, we collected baseline and follow-up data regarding demographics, pulmonary function tests and diffusion-capacity using a body plethysmograph as per ATS/ERS guidelines (Graham et al., 2019) and the gender-age-physiology (GAP) score and index (taking into account forced vital capacity (FVC), single breath diffusing capacity for the lung for carbon monoxide (SB-DLCO), age and gender (Ley et al., 2012). The GAP index has been validated as a prediction tool for mortality in IPF patients but is often used as a surrogate for disease severity (Robbie et al., 2017).

The study was conducted in accordance with the 1964 Declaration of Helsinki and its later amendments. All patients provided written informed consent, and collection of bio-samples was registered at the German Clinical Trials Register (DRKS00000017 and DRKS00000620). The respective institutional review boards approved of the bio-sampling (Freiburg 47/06 10 Marc^h^ 2006, Hannover, #2923–2015 and #2516–2014, 2 Nov 2015).

Based on a smaller pilot study (data not shown) we estimated a significantly regulated proportion of metabolites at about 15%. Using the maximum number of targeted metabolites of the MetSizeR package (Nyamundanda et al., 2013; Billoir et al., 2015), an false discovery rate (FDR) of 0.05 and a minimum sample size of at least n = 10 and PPCA model, we calculated a necessary minimum group size of *n* = 16 per group (Supplementary Figure S1). Groups were considered as nintedanib and pirfenidone treated IPF patients (with a pre-antifibrotic and post-antifibrotic sub-group) and healthy volunteers (HV). We increased the discovery group size (IPF and HV patients) to increase statistical power to detect pathway regulation while keeping the confirmation cohort around the minimal needed sample size with *n* = 21 patients.

### 2.2 Sample preparation for metabolic/lipidomic analysis

**FIGURE 1:**
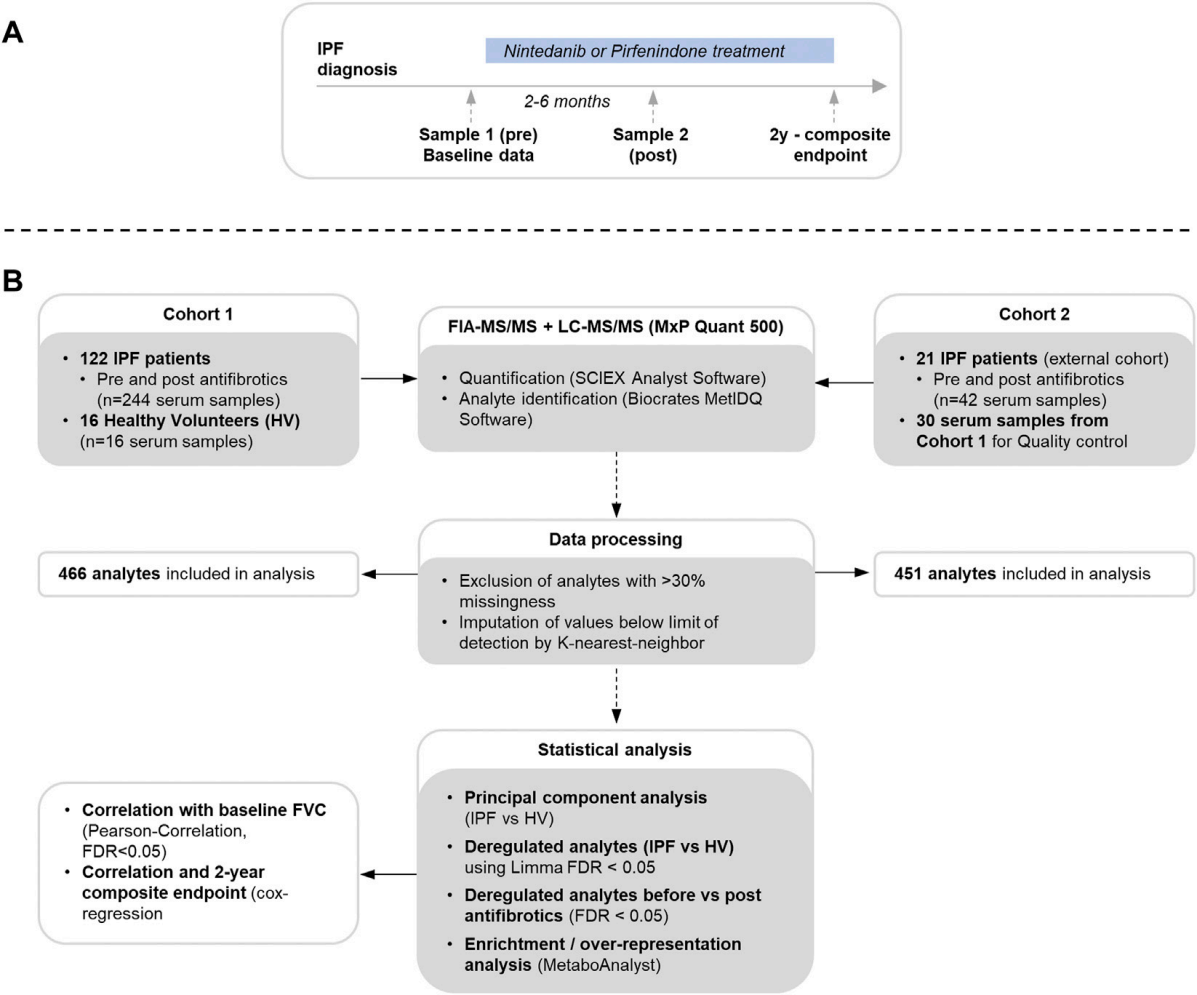
Flowchart for sample and clinical data acquisition **(A)** and metabolome measurement, data processing and statistical analysis **(B)**.

Serum samples were collected prior to initiation of antifibrotic therapy and at follow-up between 2 and 6 months after treatment start (Figure 1A). Blood samples were rested for 20 min with subsequent centrifugation. The samples were aliquoted and stored at -80°C until performance of the metabolomic studies as recommended by published protocols (Beckonert et al., 2007).

Serum metabolites were analyzed using an SCIEX 5500 QTrap mass spectrometer (SCIEX, Darmstadt, Germany) with use of the MxP Quant 500 kit (Biocrates Life Sciences AG, Innsbruck, Austria) as per manufacturer’s protocol (https://biocrates.com/mxp-quant-500-kit, accessed 14 Dec 2021) using 10 µl of the sample as previously described (Ringseis et al., 2021) with details in the supplementary material. An overview of the study flow is shown in Figure 1B.

### 2.3 Data analysis

Following metabolite measurements, metabolites with a measurement of >30% below the lower limit of detection were excluded from further analysis since high missingness limits the validity of data imputation (Faquih et al., 2020). For the remaining metabolites, values below the lower limit of detection were imputed using a k-nearest neighbor with k = 10 imputation steps on observations with variable pre-selection method (Faquih et al., 2020) using the provided R function by Faquih et al. (Faquih, 2020) assuming data to be missing completely at random. Homogeneity after imputation was visually ascertained.

For patient characteristics, categorical data was expressed as number (percentage) and compared via Chi^2^ test and continuous data was expressed as median with interquartile range (IQR) and compared via rank-sum-test. A two-tailed *p*-value of <0.05 was considered statistically significant.

Deregulation of metabolites between HV and IPF patients (at baseline) and changes following antifibrotic therapy (post vs. pre) were calculated using the R limma package (Ritchie et al., 2015) with pair-wise comparison and adjustment for multiple comparison using the FDR with an FDR <0.05 considered to be statistically significant. Heatmaps of deregulated metabolite sets were produced using the R pheatmap package with scaling and centering (to a mean of 0 with a variance of 1). Hierarchical clustering using Euclidean distances were applied to rows and columns (ward.D method). To conduct an enrichment and overrepresentation pathway analysis of the deregulated analytes, KEGG IDs were retrieved from the annotation tables provided by Biocrates Life Sciences AG and analyzed via MetaboAnalyst 5.0 (Pang et al., 2021). Principal component analysis was performed using the deregulated analytes between IPF and healthy volunteers using the R prcomp package and were visualized using the pca3d package.

Baseline metabolite concentrations were correlated with baseline GAP index and annualized FVC decline during follow-up calculating the Pearson correlation coefficient. To analyze impact on outcome, a composite endpoint including death, FVC decline of ≥10% from baseline or DLCO decline of ≥15% was calculated. Baseline metabolite concentrations in IPF patients were dichotomized by median and hazard ratios (HR) for the composite endpoint were calculated via cox-regression modeling using the metabolite median and GAP index. Identified hierarchical clusters from deregulated analytes following antifibrotic therapy were also analyzed with respect to the composite endpoint using the same cox-regression model.

## 3 Results

### 3.1 Patient cohorts

In the first cohort from Germany, 122 patients with IPF and 16 healthy volunteers of similar age (median age 65 years; 38% female) were included in the study (Table 1). Median age was 72 years in IPF vs. 65 years (HV) with a median FVC of 68% of predicted at time of initiation of antifibrotic therapy (55% nintedanib, 45% pirfenidone).

**TABLE 1:** Demographics of study cohorts at start of antifibrotic therapy.

Characteristics	Healthy volunteers (n = 16)	IPF cohort 1 (n = 122)	IPF Cohort 2 (n = 21)	*p*-value (Cohort 1 vs. Cohort 2)
Antifibrotic treatment, n (% of IPF)	-	122 (100)	21 (100)	
Nintedanib	-	67 (55)	15 (71)	
Pirfenidone	-	55 (45)	6 (29)	
Age (years), median (IQR)	65 (61–73)^a^	72 (65–76)	65 (62–73)	0.042
Female gender, n (%)	6 (38)	27 (22)	2 (10)	0.193
Forced vital capacity at baseline (% predicted), median (IQR)	118 (105–132)	68 (57–80)	83 (72–94)	<0.001
GAP Index	-			0.001
I		34 (28)	12 (67)	
II		65 (53)	6 (33)	
III		23 (19)	-	
Comorbidities, n (%)	-			
Coronary artery disease		38 (31)	9 (42)	0.400
Diabetes mellitus		26 (21)	7 (33)	0.302
Arterial Hypertension		47 (38.5)	13 (62)	0.080
Chronic kidney disease		5 (4)	0	0.328
Chronic pulmonary obstructive disease		2 (10)	12 (10)	0.890
Previous smoking history		73 (60)	15 (71)	0.513

GAP, Gender; Age, and Physiology index; IQR, interquartile range; IPF, idiopathic pulmonary fibrosis.

a Kruskal Wallis test between healthy volunteers and IPF, cohorts *p* = 0.023.

In the second cohort from Spain, 21 patients with IPF were included (median age 65 years, median baseline FVC 83% of predicted). The majority was started on nintedanib (71%) and 29% on pirfenidone. Notably, the overall disease severity measured by GAP score/index was higher in the first cohort (*p* < 0.001) while the comorbidity profile was similar.

### 3.2 Metabolite/lipid detection

In the first cohort, a total of 262 samples were measured. After discarding samples with >30% values below the lower limit of detection, a total of 466 analytes (393 lipids; 73 small molecules) were considered for further analysis, consisting of 12 lipid and 13 small molecule classes. In the second cohort, a total of 42 samples were measured with a total of 451 considered analytes (377 lipids; 74 small molecules). The dataset of cohort 1 and 2 is available online (Seeliger et al., 2022).

### 3.3 Deregulated analytes between healthy volunteers and idiopathic fibrosis patients

**FIGURE 2:**
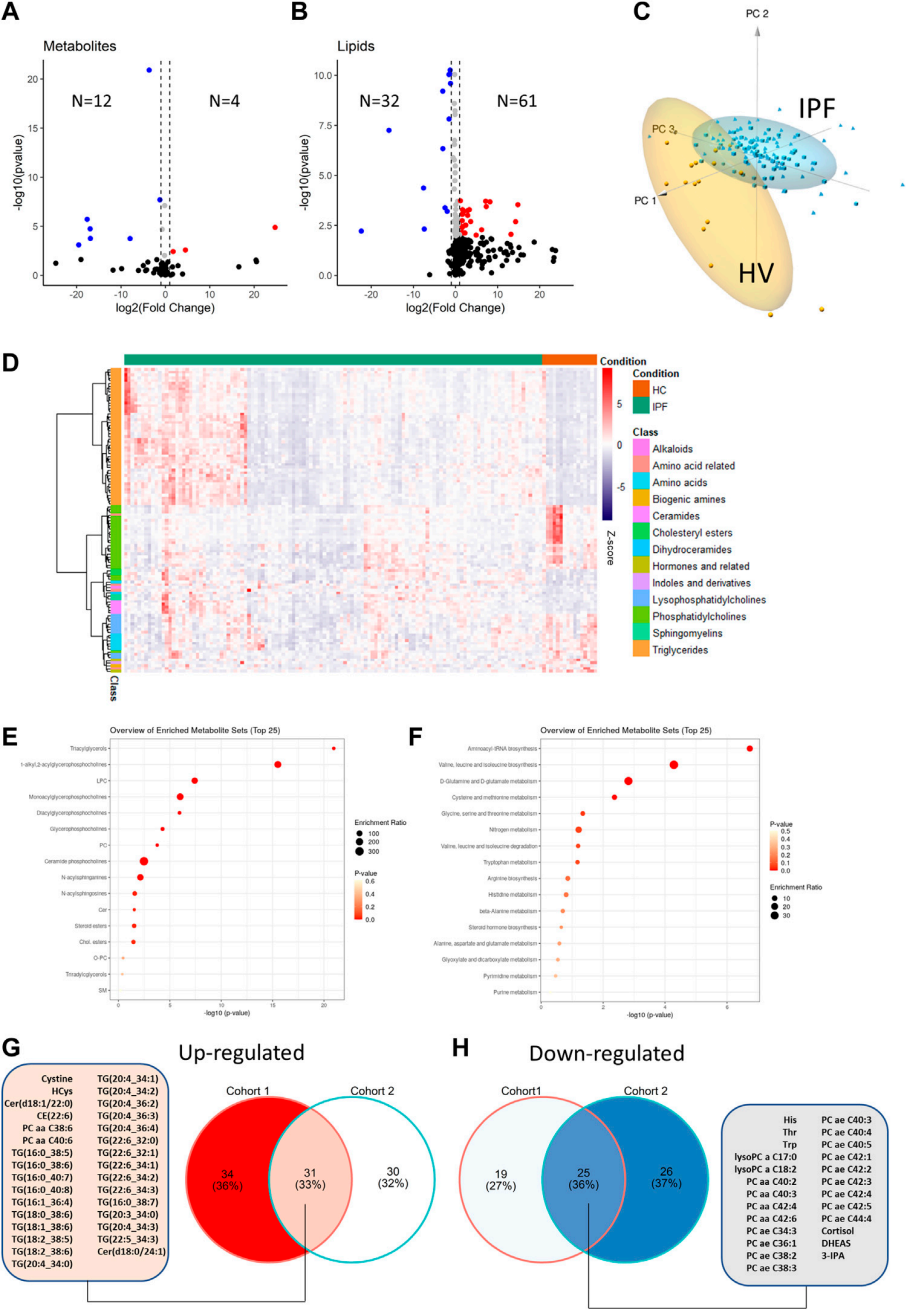
Comparison of metabolite differential abundance between patients with idiopathic pulmonary fibrosis and healthy volunteers. Log2-Fold changes were plotted against -log10 (*p*-value) of cohort 1 vs. IPF for small molecules **(A)** and lipids **(B)** as volcano-plots with numbers of significantly (FDR<0.05) up or down-regulated analytes indicated. Deregulated analytes were scaled and plotted as a 3 days principal component analysis with high lighting of IPF vs. healthy volunteers (HV) clusters **(C)**. De-regulated analytes are plotted as a heatmap with hierarchical clustering of analytes (row-wise) and visualization of abundance by Z-score **(D)**. KEGG IDs (small molecules) or compound names (lipids) were analyzed for pathway enrichment with resulting enrichment ratios and *p*-values plotted for small molecule pathways **(E)** and lipid pathways **(F)**. The overlap between de-regulated analytes from the IPF cohort 1 and cohort 2 are shown as Venn diagram in **(G)** and **(H)** with a list of common de-regulated analytes.

In a first step, samples from IPF patients in the first cohort before initiation of antifibrotic therapy were compared to healthy volunteers. For small molecules, a total of 12 analytes were significantly down-regulated in IPF (defined as FDR <0.05) and 4 were up-regulated (Supplementary Table S1; Figure 2A). For lipids, there were 32 analytes down-regulated and 61 up-regulated (Supplementary Table S1, Figure 2B).

On the basis of the de-regulated analytes, clear discrimination between HV and IPF patients was possible by principal component analysis (Figure 2C). The differential regulation of lipid and small molecule subclasses is shown in the heatmap (Figure 2D). Foremost, triglycerides were upregulated in IPF while lysophosphatidylcholines and phosphatidylcholines were down-regulated. The enriched lipid subclasses are shown in Figure 2E. Enrichment analysis of small molecules using KEGG IDs showed regulation of Aminoacyl-tRNA biosynthesis, Valin-leucine and isoleucine biosynthesis and d-Glutamine and d-Glutamate metabolism (Figure 2F).

To validate these findings, we compared the IPF patients from cohort 2 with the HVs and found similar results (51 down-regulated; 61 up-regulated) with significant overlap (Supplementary Table S2, in particular for the above-mentioned regulated lipid classes (Figures 2G,H). Notably, only three of these regulated analytes were significantly regulated by gender (Leucine, Betaine and Sphingomyelin C18:0).

### 3.4 Correlation of analytes with clinical features and survival (IPF cohort 1)

We calculated the Pearson correlation coefficients between the baseline GAP points (which are used to calculate the GAP index) and found an FDR corrected significant correlation in 94 metabolites (Supplementary Table S3). Nintety-one of 97 correlated analytes were lipids (97%) of which the majority were triglycerides (79/94, 87%), Lysophosphatidylcholines and Diglycerides. For lipids, the correlation was always negative, meaning a higher baseline lipid concentration was associated with fewer GAP points (indicating overall better performance status and prognosis).

Of note, no significant correlation between annualized decline of forced vital capacity was found for any of the analytes.

**FIGURE 3:**
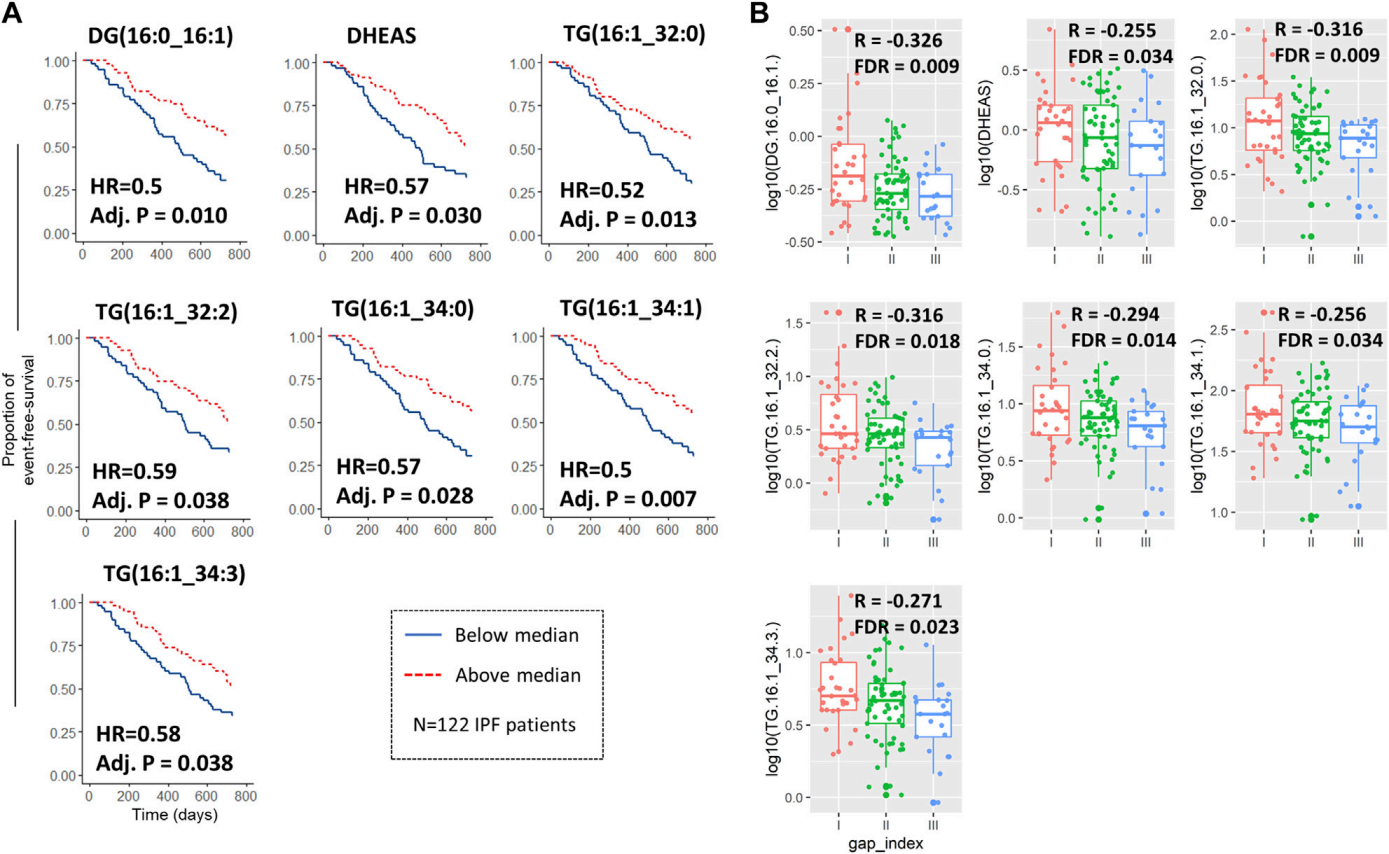
Metabolites and lipids both associated with survival/composite endpoint and baseline GAP score. Kaplan-Meier curves with adjusted hazard ratios and *p*-values for the 7 analytes which were significantly associated with both the 2-years composite endpoint when dichotomized by median and also with Gender, Age, and Physiology (GAP) score at baseline **(A)**. Log10 transformed analyte abundance was plotted against the resulting GAP indices at baseline as box-jitter-plots with associated Person correlation coefficients (between abundance and GAP score) and false discovery rate **(B)**. All IPF patients of cohort 1 were included in the analysis (n = 122).

We then dichotomized the baseline analyte concentration by the median of the IPF cohort 1 and fitted a cox-regression model for the median cut-off for each analyte adjusting for age and baseline FVC and gender. There were 16 significant analytes on cox-regression, again with the majority being lipids (Table 2). Seven analytes were both significantly correlated with survival (Figure 3A) and baseline GAP points (Figure 3B), of which 5 were triglycerides, 1 diacylglyceride (Diacylglyceride (16:0/16:1) and one small molecule (Dehydroepiandrosterone sulfate [DHEAS]). For all analytes, below-median analyte concentrations were associated with worse survival in IPF patients.

**TABLE 2:** List of analytes significantly associated with the 2 years composite endpoint of FVC decline >10%, DLCO decline >15% or death.

Analyte	Adj. Hazard Ratio	Adj. *p*-Value	Class
Diacylglyceride (16:0_16:1)	0.51	0.010	Diglycerides
Diacylglyceride (18:1_18:3)	1.73	0.041	Diglycerides
Octadecenoic acid	0.54	0.018	Fatty acids
Dehydroepiandrosterone sulfate	0.57	0.031	Hormones and related
Lysophosphatidylcholine a C18:0	0.48	0.006	Lysophosphatidylcholines
Lysophosphatidylcholine a C16:1	0.52	0.013	Lysophosphatidylcholines
Hypoxanthine	0.60	0.044	Nucleobases and related
Phosphatidylcholine ae C42:5	1.74	0.036	Phosphatidylcholines
Phosphatidylcholine ae C44:6	1.70	0.039	Phosphatidylcholines
Triacylglyceride (16:1_34:1)	0.50	0.007	Triglycerides
Triacylglyceride (16:1_32:0)	0.52	0.013	Triglycerides
Triacylglyceride (16:1_34:0)	0.57	0.028	Triglycerides
Triacylglyceride (16:1_34:3)	0.58	0.038	Triglycerides
Triacylglyceride (16:1_32:2)	0.59	0.038	Triglycerides
Triacylglyceride (17:1_34:1)	0.60	0.043	Triglycerides
Choline	0.51	0.010	Vitamins and cofactors

### 3.5 Metabolite/lipid changes following antifibrotic therapy

Following antifibrotic therapy of median 8 (5–16) weeks, another set of serum samples were collected and remeasured. Interestingly, we did not observe any deregulated analytes following treatment with pirfenidone in both cohorts.

**FIGURE 4:**
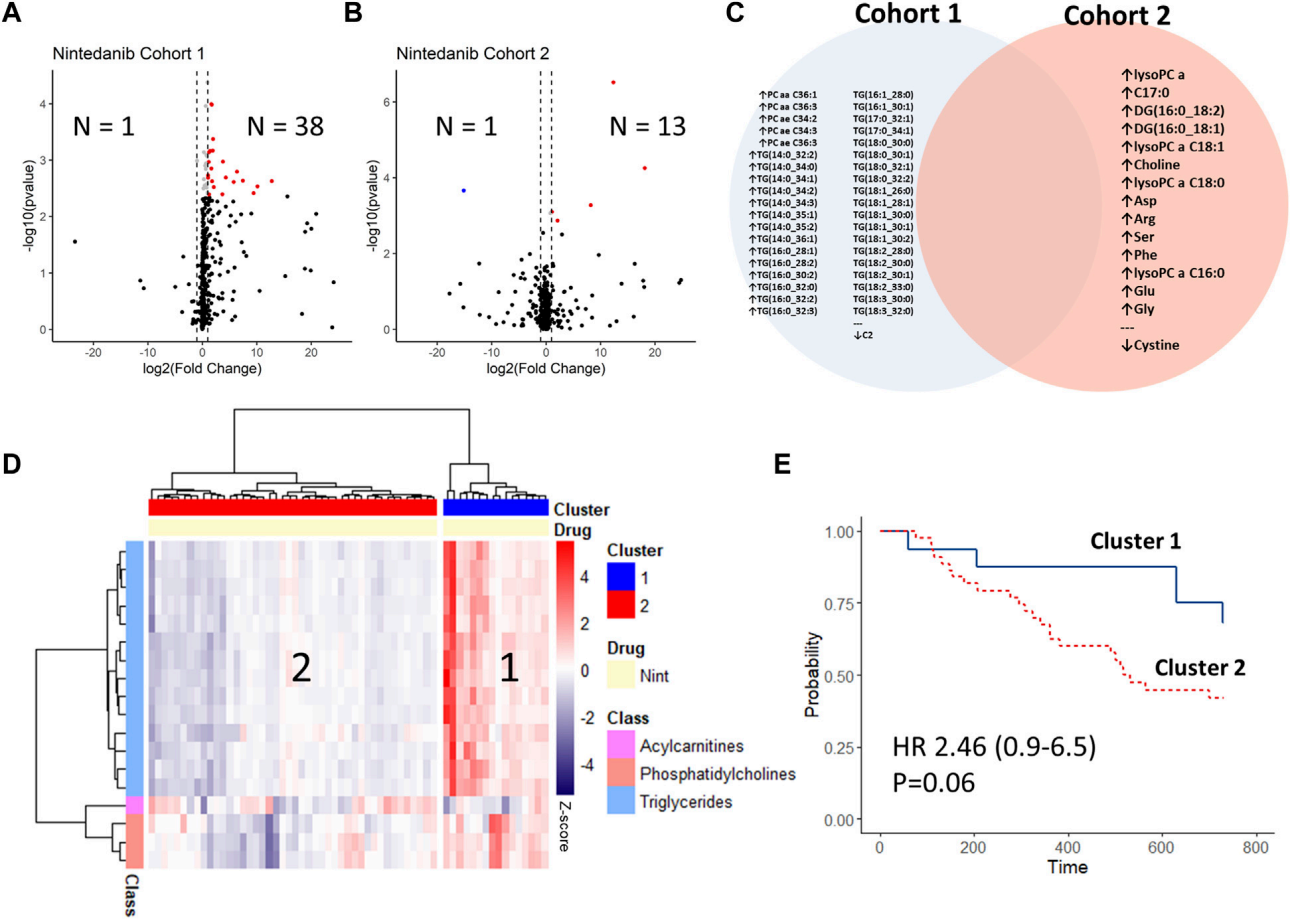
Changes in longitudinal analysis before vs. after initiation of antifibrotic treatment with nintedanib. Log2-Fold changes were plotted against -log10 (*p*-value) of cohort 1 **(A)** and cohort 2 **(B)** as volcano-plots with numbers of significantly (FDR<0.05) up or down-regulated analytes indicated (sample after treatment vs. baseline). The deregulated analytes in both cohorts are shown in **(C)**, with no overlap between the cohorts. Delta-values (sample after treatment vs. baseline) of cohort 1 were calculated per patient and changes between the de-regulated analytes were plotted via heat-map with hierarchical clustering (ward.D method) **(D)**. The resulting patient clusters were then compared via Kaplan-Meier curves and adjusted cox-regression modelling (for GAP-index) **(E)**.

Following treatment with nintedanib in the first IPF cohort, there were 38 up-regulated analytes and 1 down-regulated analyte, all of which were lipids (33 triglycerides, 5 phosphatidylcholines and 1 acylcarnitine (Supplementary Table S4, Figure 4A). In the second cohort there were 13 up-regulated analytes and 1 down-regulated analyte (Supplementary Table S4, Figure 4B), but there was no overlap between the deregulated analytes between the cohorts (Figure 4C). Also, only one of the deregulated analytes following nintedanib treatment was mutually deregulated between IPF at baseline and HV (Phosphatidylcholine ae C34:3).

We then calculated the changes in metabolite concentrations between baseline and the follow-up samples on the subset of analytes which were deregulated. Using hierarchical clustering, we found that the subgroup of patients who had an increase in deregulated analyte concentrations after nintedanib (mostly triglycerides) had a trend towards better survival, albeit missing statistical significance (HR 2.46 [CI 0.93–6.48]; *p* = 0.06) (Figures 4D,E).

### 3.6 Quality control

To account for compatibility, 30 samples from cohort 1 were re-measured alongside with cohort 2, with good correlation between the re-measured samples and a median ratio between analytes of 1.03 (IQR 0.9–1.18) (Supplementary Figure S2).

## 4 Discussion

Recent data from the cancer field and pulmonary fibrosis suggest a major role of metabolomic changes in both disease pathogenesis and treatment. On this background we got interested in the metabolome of IPF patients and whether pirfenidone or nintedanib induce any metabolic changes. We comprehensively studied the metabolome and lipidome of two IPF cohorts comprising 143 patients and age-matched healthy volunteers.

The serum metabolome of healthy volunteers differed considerably from IPF patients in both studied cohorts. One-hundred-nine of the 466 (23.4%) included analytes were differentially abundant in IPF in the first cohort. Compared to healthy volunteers we found 44 analytes downregulated and 65 analytes upregulated. Most impressively were the changes in the lipidome. Among the 44 downregulated metabolites in serum of IPF patients were 8 amino acids or amino acid related metabolites, 4 biogenic amines or hormones, but the majority of the significantly downregulated metabolites were lipids including 9 (20.5%) lysophosphatidylcholines, and 23 (52%) phosphatidylcholines. On the other hand, 65 metabolites were significantly upregulated and these were again mostly composed of lipids (94%) but with different subclasses. The majority of the up-regulated lipids were triglyceride (75%) and some were ceramides (8%), sphingomyelins or cholesteryl esters. A similar metabolome profile in IPF patients was also described by Yan et al., although the study cohort was substantially smaller and consisted only of 22 IPF patients (Yan et al., 2017). In addition, other studies reported also on changes in the metabolome of IPF patients but included only small cohorts consisting of less than 30 patients or no healthy controls and without serial measurements (Rindlisbacher et al., 2018; Nambiar et al., 2021a; Nambiar et al., 2021b). In line with other reports, we did not find major changes in metabolome associated with age or gender. Thus, we observed vast changes in the metabolome and lipidome of IPF patients.

Changes in metabolome are highly disease specific and were reported to serve as robust biomarkers (Trezzi et al., 2015). The finding of multiple different triglycerides upregulated in IPF attracted our attention. Tryglycerides are abundant circulating lipids and are stored in droplets formed in the endoplasmic reticulum (ER) (Nambiar et al., 2021b) where they may induce the expression of ER stress markers (Kim et al., 2007). ER stress has been linked to misfolded gene production in type II alveolar epithelial cells leading to pulmonary fibrosis via multiple mechanisms, including M2 macrophage polarization and alveolar epithelial cell apoptosis (Burman et al., 2018). High-fat diets were also shown to exacerbate pulmonary fibrosis in mice via modulation of ER stress (Chu et al., 2019). An upregulation of certain triglycerides has been described in progressing compared to stable IPF patients (Nambiar et al., 2021b) whilst we found elevated levels in IPF vs. HV. Contrarily to Nambiar et al., we found within IPF patients that lower baseline triglyceride levels were associated with poor prognosis. The association found in our data may also be due to effects of pulmonary cachexia in patients with more advanced disease (Luppi et al., 2021). The mechanistic involvement of the individual triglycerides found is not clear and more research focusing on triglyceride effects in pulmonary fibrosis models is needed.

Interestingly also in other types of organ fibrosis an increase in triglycerides was noted such as chronic kidney disease and fibrotic liver diseases (Chen et al., 2017; Monteillet et al., 2018; Harzandi et al., 2021). In addition, an increase in triglycerides was also observed in several murine models of organ fibrosis (Harzandi et al., 2021; Weckerle et al., 2021). In contrast, patients with cancer including lung malignancies show a down-regulation of triglycerides, which is associated with poor outcome (Siemianowicz et al., 2000). It was speculated that an increase in triglycerides may derive from increased cell death and injury, while in cancer cells proliferation consumes triglycerides, a major constituent of cells and energy provider. We also found ceramides up-regulated in IPF. Ceramides are important in epithelial barrier integrity and were also reported to be up-regulated in airway diseases such as COPD (Teichgräber et al., 2008; Bowler et al., 2015; Cruickshank-Quinn et al., 2018). Thus, increase in multiple types of triglycerides and ceramide is a hallmark of IPF and may be related to epithelial pathology.

Lysophosphatidylcholines and phosphatidylcholines were on the other hand down-regulated in serum of IPF patients. A major constituent of surfactant are phosphatidylcholines and it is thought that decrease of these lipoproteins in IPF and COPD is caused by decreased surfactant protein production by reduced numbers of alveolar epithelial type II cells (Cruickshank-Quinn et al., 2018). Interestingly, different lysophosphatidylcholines were found to be upregulated (as opposed to the downregulation in both our IPF cohorts) in two other publications. Rindlisbacher and coworkers found one unspecified Lysopgopshatidylcholine which was upregulated in 10 stable IPF compared to HV. In another cohort of IPF patients and HV published in abstract form, LysoPC(20:3) was reportedly upregulated while it was downregulated in our cohort. Given only minimal patient information being available for the second study (especially antifibrotic medication) and several LysoPCs were upregulated following nintedanib treatment (Table S4) this may potentially explain the discrepancy. Either way, LysoPCs seem to be involved in IPF pathogenesis. LysoPCs serve as precursor molecules in the production of lysophosphatidic acid (LPA) via ectonucleotide pyrophosphatase-phosphodiesterase 2 (ENPP2), or Autotaxin (ATX) (Ninou et al., 2018). LPA mediates its effects via a range of receptors, most importantly LPAR1, contributing to profibrotic fibroblast activation (Tager et al., 2008), TGF-ß activation (Xu et al., 2009; Huang et al., 2013) and endothelial permeability promoting inflammation (Ninou et al., 2018). ATX was shown to be upregulated in bronchoalveolar lavage fluid in bleomycin models (Oikonomou et al., 2012) and its inhibition ameliorated LPA levels and pulmonary fibrosis in bleomycin models (Oikonomou et al., 2012; Kato et al., 2016; Desroy et al., 2017). These findings served as rationale for clinical trials with autotaxin inhibitors in IPF (Maher et al., 2019). Despite these effects of LPA and other known effects of LysoPC as their precursors, correlation with circulating LysoPC levels are unclear and in some diseases even inverse correlations have been described (Law et al., 2019), rendering direct measurements of LPA or autotaxin activity more suitable for correlations in IPF.

Changes in amino acids are also of high interest. We and others found l-glutamine highly upregulated (Log2 fold-change 105) in IPF. TGF-β, upregulated in fibrosis, was shown to induce glutaminolysis in lung fibroblasts and consecutively leads to increased collagen production (Bernard et al., 2018; Hamanaka et al., 2019).

Our cohort was large enough to allow for survival and outcome analyses. Using cox-regression modeling we found 16 metabolites associated with a composite endpoint of time to disease progression or death. Among the disease progression associated metabolites were lyso-phosphatidylcholine, choline and triacylglyceride (16:1_34:1). None of these metabolites were differentially expressed in the comparison of healthy volunteers and IPF.

Our study included also serial measurements of patients in whom treatment with either pirfenidone or nintedanib was initiated. A second serum sample was obtained 8 weeks after therapy start. While we did not find any significant metabolite changes with pirfenidone treatment, significant changes were induced by nintedanib. Unexpectedly most of the differentially expressed metabolites were up-regulated and were not related to the above described changes in serum metabolome of IPF patients versus healthy volunteers. Of interest, multiple studies reported metabolic changes with tyrosine kinase inhibitor (TKI) treatment. Growing evidence indicates major changes in metabolome with imatinib, a TKI targeting platelet-derived growth factor (PDGF) receptors, used for treatment in chronic myeloid leukemia (Póvoa et al., 2021). Likewise, *in-vitro* experiments with a macrophage cell line indicated that SU1498, a TKI blocking VEGF-R signaling, induced upregulation of triglycerides and a decrease in glycerophosphocholine (Mesti et al., 2014). These data suggest that the metabolic changes observed with nintedanib are rather a direct drug-effect and not related to modulation of disease associated metabolic changes. Nevertheless, we observed that the described upregulation of triglycerides by nintedanib treatment was only present in a subset of the patients, while others did not show this finding when serum samples prior and during nintedanib treatment were compared. Also, the deregulated analytes were different between the two IPF cohorts, potentially owing to the milder disease extent in the second cohort. Interestingly the group of patients with nintedanib induced changes in their metabolome had the best outcome results and showed a significantly longer time to disease progression or death compared to patients with no treatment induced metabolic changes. These data suggest that treatment efficacy may differ between patient subsets and metabolic changes and especially an increase in triglycerides may serve as a biomarker for treatment response.

Our study has significant limitations. Since we found metabolites that correlated with disease severity, the milder disease extent in the second cohort may partially explain incongruent findings between the IPF cohorts and may hamper comparability. Further, the overall milder disease in cohort 2 and smaller group size prevented us from running correlations with clinical features and analyte concentrations in this cohort. The analytes identified to correlate with both survival and baseline GAP score showed a rather low correlation, rendering their biological relevance subject to further studies. Metabolomic measurements are subject to a variety of confounders, including environmental factors (Lu et al., 2017). The cohort 1 and 2 were not measured on the same day, although a number of samples from cohort 1 was repeatedly measured on the same batch as cohort 2, with only moderate variances in metabolite levels. Dietary differences between patients were not assessed but may have influenced the results. Likewise, due to frequent adverse events, the overall adherence to antifibrotic therapy may vary broadly and some patients in these cohorts may not have taken their medication at the time of sampling (B103, 2019). Importantly, the high throughput lipidomic technology used herein does not allow for reliable identification of exact lipid structures (i.e. TG (16:0_34:2) allows for 6 potential isomers). Follow-up investigations for candidate lipids thorough to classify these lipids in detail are needed (Liebisch et al., 2013). We acknowledge our results at this stage are merely hypothesis generating, but given the lack of treatment response biomarkers in IPF, exploratory analyses seem warranted.

In conclusion, we report major changes in metabolites in two independent cohorts testing a large number of patients. Several metabolites are associated with poor outcome. In summary, specific lipidic metabolite signatures may serve as biomarkers for disease progression or favorable treatment response to nintedanib.

## Data Availability

The datasets for this study can be found online at https://zenodo.org/record/6394924 (Seeliger et al., 2022).

## References

[B1] B103 (2019). ILD: THERAPY. American thoracic society 2019 international conference. Dallas, TX: American Thoracic Society, 052019.

[B2] Beckonert O. Keun H. C. Ebbels T. M. D. Bundy J. Holmes E. Lindon J. C. (2007). Metabolic profiling, metabolomic and metabonomic procedures for NMR spectroscopy of urine, plasma, serum and tissue extracts. Nat. Protoc. 2, 2692–2703. 10.1038/nprot.2007.376 18007604

[B3] Bernard K. Logsdon N. J. Benavides G. A. Sanders Y. Zhang J. Darley-Usmar V. M. (2018). Glutaminolysis is required for transforming growth factor-β1-induced myofibroblast differentiation and activation. J. Biol. Chem. 293, 1218–1228. 10.1074/jbc.RA117.000444 29222329 PMC5787800

[B4] Billoir E. Navratil V. Blaise B. J. (2015). Sample size calculation in metabolic phenotyping studies. Brief. Bioinform. 16, 813–819. 10.1093/bib/bbu052 25600654

[B5] Bowler R. P. Jacobson S. Cruickshank C. Hughes G. J. Siska C. Ory D. S. (2015). Plasma sphingolipids associated with chronic obstructive pulmonary disease phenotypes. Am. J. Respir. Crit. Care Med. 191, 275–284. 10.1164/rccm.201410-1771OC 25494452 PMC4351578

[B6] Burman A. Tanjore H. Blackwell T. S. (2018). Endoplasmic reticulum stress in pulmonary fibrosis. Matrix Biol. 68-69, 355–365. 10.1016/j.matbio.2018.03.015 29567124 PMC6392005

[B7] Chen D-Q. Chen H. Chen L. Vaziri N. D. Wang M. Li X. R. (2017). The link between phenotype and fatty acid metabolism in advanced chronic kidney disease. Nephrol. Dial. Transpl. 32, 1154–1166. 10.1093/ndt/gfw415 28339984

[B8] Chu S. G. Villalba J. A. Liang X. Xiong K. Tsoyi K. Ith B. (2019). Palmitic acid-rich high-fat diet exacerbates experimental pulmonary fibrosis by modulating endoplasmic reticulum stress. Am. J. Respir. Cell Mol. Biol. 61, 737–746. 10.1165/rcmb.2018-0324OC 31461627 PMC6890409

[B9] Chung Y-L. Griffiths J. R. (2007). Using metabolomics to monitor anticancer drugs. Ernst Scher. Found. Symp. Proc. 4, 55–78. 10.1007/2789_2008_089 18811053

[B10] Conte E. Gili E. Fagone E. Fruciano M. Iemmolo M. Vancheri C. (2014). Effect of pirfenidone on proliferation, TGF-β-induced myofibroblast differentiation and fibrogenic activity of primary human lung fibroblasts. Eur. J. Pharm. Sci. 58, 13–19. 10.1016/j.ejps.2014.02.014 24613900

[B11] Cruickshank-Quinn C. I. Jacobson S. Hughes G. Powell R. L. Petrache I. Kechris K. (2018). Metabolomics and transcriptomics pathway approach reveals outcome-specific perturbations in COPD. Sci. Rep. 8, 17132. 10.1038/s41598-018-35372-w 30459441 PMC6244246

[B12] Desroy N. Housseman C. Bock X. Joncour A. Bienvenu N. Cherel L. (2017). Discovery of 2-2-Ethyl-6-4-2-(3-hydroxyazetidin-1-yl)-2-oxoethylpiperazin-1-yl-8-methylimidazo1, 2-apyridin-3-ylmethylamino-4-(4-fluorophenyl)thiazole-5-carbonitrile (GLPG1690), a first-in-class Autotaxin inhibitor undergoing clinical evaluation for the treatment of idiopathic pulmonary fibrosis. J. Med. Chem. 60, 3580–3590. 10.1021/acs.jmedchem.7b00032 28414242

[B13] Epstein Shochet G. Wollin L. Shitrit D. (2018). Fibroblast-matrix interplay: Nintedanib and pirfenidone modulate the effect of IPF fibroblast-conditioned matrix on normal fibroblast phenotype. Respirology 23, 756–763. 10.1111/resp.13287 29532550

[B14] Faquih T. van Smeden M. Luo J. le Cessie S. Kastenmuller G. Krumsiek J. (2020). A workflow for missing values imputation of untargeted metabolomics data. Metabolites 10, E486. 10.3390/metabo10120486 33256233 PMC7761057

[B15] Faquih T (2020). tofaquih/imputation_of_untargeted_metabolites. Zenodo: Offical Release.

[B16] Gaugg M. T. Engler A. Bregy L. Nussbaumer-Ochsner Y. Eiffert L. Bruderer T. (2019). Molecular breath analysis supports altered amino acid metabolism in idiopathic pulmonary fibrosis. Respirology 24, 437–444. 10.1111/resp.13465 30681243

[B17] Graham B. L. Steenbruggen I. Miller M. R. Barjaktarevic I. Z. Cooper B. G. Hall G. L. (2019). Standardization of spirometry 2019 update. An official American thoracic society and European respiratory society technical statement. Am. J. Respir. Crit. Care Med. 200, e70–e88. 10.1164/rccm.201908-1590ST 31613151 PMC6794117

[B18] Hamanaka R. B. O'Leary E. M. Witt L. J. Tian Y. Gokalp G. A. Meliton A. Y. (2019). Glutamine metabolism is required for collagen protein synthesis in lung fibroblasts. Am. J. Respir. Cell Mol. Biol. 61, 597–606. 10.1165/rcmb.2019-0008OC 30973753 PMC6827066

[B19] Harzandi A. Lee S. Bidkhori G. Saha S. Hendry B. M. Mardinoglu A. (2021). Acute kidney injury leading to CKD is associated with a persistence of metabolic dysfunction and hypertriglyceridemia. iScience 24, 102046. 10.1016/j.isci.2021.102046 33554059 PMC7843454

[B20] Hostettler K. E. Zhong J. Papakonstantinou E. Karakiulakis G. Tamm M. Seidel P. (2014). Anti-fibrotic effects of nintedanib in lung fibroblasts derived from patients with idiopathic pulmonary fibrosis. Respir. Res. 15, 157. 10.1186/s12931-014-0157-3 25496490 PMC4273482

[B21] Huang L. S. Fu P. Patel P. Harijith A. Sun T. Zhao Y. (2013). Lysophosphatidic acid receptor-2 deficiency confers protection against bleomycin-induced lung injury and fibrosis in mice. Am. J. Respir. Cell Mol. Biol. 49, 912–922. 10.1165/rcmb.2013-0070OC 23808384 PMC3931116

[B22] Jee A. S. Sahhar J. Youssef P. Bleasel J. Adelstein S. Nguyen M. (2019). Review: Serum biomarkers in idiopathic pulmonary fibrosis and systemic sclerosis associated interstitial lung disease - frontiers and horizons. Pharmacol. Ther. 202, 40–52. 10.1016/j.pharmthera.2019.05.014 31153954

[B23] Jin J. Togo S. Kadoya K. Tulafu M. Namba Y. Iwai M. (2019). Pirfenidone attenuates lung fibrotic fibroblast responses to transforming growth factor-β1. Respir. Res. 20, 119. 10.1186/s12931-019-1093-z 31185973 PMC6558902

[B24] Kato K. Ikeda H. Miyakawa S. Futakawa S. Nonaka Y. Fujiwara M. (2016). Structural basis for specific inhibition of Autotaxin by a DNA aptamer. Nat. Struct. Mol. Biol. 23, 395–401. 10.1038/nsmb.3200 27043297

[B25] Kim D-S. Jeong S-K. Kim H-R. Chae S. W. Chae H. J. (2007). Effects of triglyceride on ER stress and insulin resistance. Biochem. Biophys. Res. Commun. 363, 140–145. 10.1016/j.bbrc.2007.08.151 17868644

[B26] King T. E. Bradford W. Z. Castro-Bernardini S. Fagan E. A. Glaspole I. Glassberg M. K. (2014). A phase 3 trial of pirfenidone in patients with idiopathic pulmonary fibrosis. N. Engl. J. Med. 370, 2083–2092. 10.1056/NEJMoa1402582 24836312

[B27] Law S-H. Chan M-L. Marathe G. K. Parveen F. Chen C. H. Ke L. Y. (2019). An updated review of lysophosphatidylcholine metabolism in human diseases. Int. J. Mol. Sci. 20, E1149. 10.3390/ijms20051149 30845751 PMC6429061

[B28] Lederer D. J. Martinez F. J. (2018). Idiopathic pulmonary fibrosis. N. Engl. J. Med. 378, 1811–1823. 10.1056/NEJMra1705751 29742380

[B29] Ley B. Ryerson C. J. Vittinghoff E. Ryu J. H. Tomassetti S. Lee J. S. (2012). A multidimensional index and staging system for idiopathic pulmonary fibrosis. Ann. Intern. Med. 156, 684–691. 10.7326/0003-4819-156-10-201205150-00004 22586007

[B30] Liebisch G. Vizcaíno J. A. Köfeler H. Trotzmuller M. Griffiths W. J. Schmitz G. (2013). Shorthand notation for lipid structures derived from mass spectrometry. J. Lipid Res. 54, 1523–1530. 10.1194/jlr.M033506 23549332 PMC3646453

[B31] Lu W. Su X. Klein M. S. Lewis I. A. Fiehn O. Rabinowitz J. D. (2017). Metabolite measurement: Pitfalls to avoid and practices to follow. Annu. Rev. Biochem. 86, 277–304. 10.1146/annurev-biochem-061516-044952 28654323 PMC5734093

[B32] Luppi F. Kalluri M. Faverio P. Kreuter M. Ferrara G. (2021). Idiopathic pulmonary fibrosis beyond the lung: understanding disease mechanisms to improve diagnosis and management. Respir. Res. 22, 109. 10.1186/s12931-021-01711-1 33865386 PMC8052779

[B33] Madama D. Martins R. Pires A. S. Botelho M. F. Alves M. G. Abrantes A. M. (2021). Metabolomic profiling in lung cancer: A systematic review. Metabolites 11, 630. 10.3390/metabo11090630 34564447 PMC8471464

[B34] Maher T. M. Kreuter M. Lederer D. J. Brown K. K. Wuyts W. Verbruggen N. (2019). Rationale, design and objectives of two phase III, randomised, placebo-controlled studies of GLPG1690, a novel autotaxin inhibitor, in idiopathic pulmonary fibrosis (ISABELA 1 and 2). BMJ Open Respir. Res. 6, e000422. 10.1136/bmjresp-2019-000422 PMC6530501 31179008

[B35] Mamazhakypov A. Schermuly R. T. Schaefer L. Wygrecka M. (2019). Lipids - two sides of the same coin in lung fibrosis. Cell. Signal. 60, 65–80. 10.1016/j.cellsig.2019.04.007 30998969

[B36] Mesti T. Savarin P. Triba M. N. Le Moyec L. Ocvirk J. Banissi C. (2014). Metabolic impact of anti-angiogenic agents on U87 glioma cells. PLoS One 9, e99198. 10.1371/journal.pone.0099198 24922514 PMC4055646

[B37] Monteillet L. Gjorgjieva M. Silva M. Verzieux V. Imikirene L. Duchampt A. (2018). Intracellular lipids are an independent cause of liver injury and chronic kidney disease in non alcoholic fatty liver disease-like context. Mol. Metab. 16, 100–115. 10.1016/j.molmet.2018.07.006 30100243 PMC6157648

[B38] Nambiar S. Clynick B. How B. S. King A. Walters E. H. Goh N. S. (2021). There is detectable variation in the lipidomic profile between stable and progressive patients with idiopathic pulmonary fibrosis (IPF). Respir. Res. 22, 105. 10.1186/s12931-021-01682-3 33836757 PMC8033725

[B39] Nambiar S. Tan D. B. A. Clynick B. Bong S. H. Rawlinson C. Gummer J. (2021). Untargeted metabolomics of human plasma reveal lipid markers unique to chronic obstructive pulmonary disease and idiopathic pulmonary fibrosis. Proteomics. Clin. Appl. 15, e2000039. 10.1002/prca.202000039 33580915

[B40] Ninou I. Magkrioti C. Aidinis V. (2018). Autotaxin in pathophysiology and pulmonary fibrosis. Front. Med. 5, 180. 10.3389/fmed.2018.00180 PMC6008954 29951481

[B41] Nyamundanda G. Gormley I. C. Fan Y. Gallagher W. M. Brennan L. (2013). MetSizeR: selecting the optimal sample size for metabolomic studies using an analysis based approach. BMC Bioinforma. 14, 338. 10.1186/1471-2105-14-338 PMC4222287 24261687

[B42] Oikonomou N. Mouratis M-A. Tzouvelekis A. Kaffe E. Valavanis C. Vilaras G. (2012). Pulmonary autotaxin expression contributes to the pathogenesis of pulmonary fibrosis. Am. J. Respir. Cell Mol. Biol. 47, 566–574. 10.1165/rcmb.2012-0004OC 22744859

[B43] Pang Z. Chong J. Zhou G. de Lima Morais D. A. Chang L. Barrette M. (2021). MetaboAnalyst 5.0: narrowing the gap between raw spectra and functional insights. Nucleic Acids Res. 49, W388–W396. 10.1093/nar/gkab382 34019663 PMC8265181

[B44] Póvoa V. M. O. Delafiori J. Dias-Audibert F. L. de Oliveira A. N. Lopes A. B. P. de Paula E. V. (2021). Metabolic shift of chronic myeloid leukemia patients under imatinib-pioglitazone regimen and discontinuation. Med. Oncol. 38, 100. 10.1007/s12032-021-01551-5 34302533

[B45] Pyne N. J. Dubois G. Pyne S. (2013). Role of sphingosine 1-phosphate and lysophosphatidic acid in fibrosis. Biochim. Biophys. Acta 1831, 228–238. 10.1016/j.bbalip.2012.07.003 22801038

[B46] Raghu G. Remy-Jardin M. Myers J. L. Richeldi L. Ryerson C. J. Lederer D. J. (2018). Diagnosis of idiopathic pulmonary fibrosis. An official ATS/ERS/JRS/ALAT clinical practice guideline. Am. J. Respir. Crit. Care Med. 198, e44–e68. 10.1164/rccm.201807-1255ST 30168753

[B47] Raghu G. Rochwerg B. Zhang Y. Garcia C. A. C. Azuma A. Behr J. (2015). An official ATS/ERS/JRS/ALAT clinical practice guideline: Treatment of idiopathic pulmonary fibrosis. An update of the 2011 clinical practice guideline. Am. J. Respir. Crit. Care Med. 192, e3–19. 10.1164/rccm.201506-1063ST 26177183

[B48] Richeldi L. Du Bois R. M. Raghu G. Azuma A. Brown K. K. Costabel U. (2014). Efficacy and safety of nintedanib in idiopathic pulmonary fibrosis. N. Engl. J. Med. 370, 2071–2082. 10.1056/NEJMoa1402584 24836310

[B49] Rindlisbacher B. Schmid C. Geiser T. Bovet C. Funke-Chambour M. (2018). Serum metabolic profiling identified a distinct metabolic signature in patients with idiopathic pulmonary fibrosis - a potential biomarker role for LysoPC. Respir. Res. 19, 7. 10.1186/s12931-018-0714-2 29321022 PMC5764001

[B50] Ringseis R. Grundmann S. M. Schuchardt S. Most E. Eder K. (2021). Limited impact of pivalate-induced secondary carnitine deficiency on hepatic transcriptome and hepatic and plasma metabolome in nursery pigs. Metabolites 11, 573. 10.3390/metabo11090573 34564388 PMC8468870

[B51] Ritchie M. E. Phipson B. Wu D. Hu Y. Law C. W. Shi W. (2015). Limma powers differential expression analyses for RNA-sequencing and microarray studies. Nucleic Acids Res. 43, e47. 10.1093/nar/gkv007 25605792 PMC4402510

[B52] Roach K. M. Castells E. Dixon K. Mason S. Elliott G. Marshall H. (2021). Evaluation of pirfenidone and nintedanib in a human lung model of fibrogenesis. Front. Pharmacol. 12, 679388. 10.3389/fphar.2021.679388 34712131 PMC8546112

[B53] Robbie H. Daccord C. Chua F. Devaraj A. (2017). Evaluating disease severity in idiopathic pulmonary fibrosis. Eur. Respir. Rev. 26, 170051. 10.1183/16000617.0051-2017 28877976 PMC9488723

[B54] Roque W. Romero F. (2021). Cellular metabolomics of pulmonary fibrosis, from amino acids to lipids. Am. J. Physiol. Cell Physiol. 320, C689–C695. 10.1152/ajpcell.00586.2020 33471621 PMC8163573

[B55] Ruwanpura S. M. Thomas B. J. Bardin P. G. (2020). Pirfenidone: Molecular mechanisms and potential clinical applications in lung disease. Am. J. Respir. Cell Mol. Biol. 62, 413–422. 10.1165/rcmb.2019-0328TR 31967851

[B56] Schmidt D. R. Patel R. Kirsch D. G. Lewis C. A. Vander Heiden M. G. Locasale J. W. (2021). Metabolomics in cancer research and emerging applications in clinical oncology. Ca. Cancer J. Clin. 71, 333–358. 10.3322/caac.21670 33982817 PMC8298088

[B57] Seeliger B. Prasse A. Carleo A. (2022). Metabolic and lipidomic data of patients with idiopathic pulmonary fibrosis and healthy volunteers.

[B58] Shea B. S. Tager A. M. (2012). Role of the lysophospholipid mediators lysophosphatidic acid and sphingosine 1-phosphate in lung fibrosis. Proc. Am. Thorac. Soc. 9, 102–110. 10.1513/pats.201201-005AW 22802282 PMC5455616

[B59] Siemianowicz K. Gminski J. Stajszczyk M. WojakoWski W. GossM. MachalskiM. (2000). Serum total cholesterol and triglycerides levels in patients with lung cancer. Int. J. Mol. Med. 5, 201–205. 10.3892/ijmm.5.2.201 10639602

[B60] Tager A. M. LaCamera P. Shea B. S. Campanella G. S. Selman M. Zhao Z. (2008). The lysophosphatidic acid receptor LPA1 links pulmonary fibrosis to lung injury by mediating fibroblast recruitment and vascular leak. Nat. Med. 14, 45–54. 10.1038/nm1685 18066075

[B61] Tedesco S. Scattolini V. Albiero M. Bortolozzi M. Avogaro A. Cignarella A. (2019). Mitochondrial calcium uptake is instrumental to alternative macrophage polarization and phagocytic activity. Int. J. Mol. Sci. 20, E4966. 10.3390/ijms20194966 31597355 PMC6801659

[B62] Teichgräber V. Ulrich M. Endlich N. Riethmuller J. Wilker B. De Oliveira-Munding C. C. (2008). Ceramide accumulation mediates inflammation, cell death and infection susceptibility in cystic fibrosis. Nat. Med. 14, 382–391. 10.1038/nm1748 18376404

[B63] Trezzi J-P. Vlassis N. Hiller K. (2015). The role of metabolomics in the study of cancer biomarkers and in the development of diagnostic tools. Adv. Exp. Med. Biol. 867, 41–57. 10.1007/978-94-017-7215-0_4 26530359

[B64] Weckerle J. Picart-Armada S. Klee S. Tom B. Andreas H. L. Wolfgang R. (2021). Mapping the metabolomic and lipidomic changes in the Bleomycin model of pulmonary fibrosis in young and aged mice. Dis. Model Mech. 15, dmm049105. 10.1242/dmm.049105 PMC8807555 34845494

[B65] Xu M. Y. Porte J. Knox A. J. Weinreb P. H. Maher T. M. Violette S. M. (2009). Lysophosphatidic acid induces alphavbeta6 integrin-mediated TGF-beta activation via the LPA2 receptor and the small G protein G alpha(q). Am. J. Pathol. 174, 1264–1279. 10.2353/ajpath.2009.080160 19147812 PMC2671359

[B66] Yan F. Wen Z. Wang R. Luo W. Du Y. Wang W. (2017). Identification of the lipid biomarkers from plasma in idiopathic pulmonary fibrosis by Lipidomics. BMC Pulm. Med. 17, 174. 10.1186/s12890-017-0513-4 29212488 PMC5719761

[B67] Yang Q. Zhang A. Miao J. Sun H. Han Y. Yan G. L. (2019). Metabolomics biotechnology, applications, and future trends: a systematic review. RSC Adv. 9, 37245–37257. 10.1039/c9ra06697g 35542267 PMC9075731

[B68] Zhao Y. D. Yin L. Archer S. Lu C. Zhao G. Yao Y. (2017). Metabolic heterogeneity of idiopathic pulmonary fibrosis: a metabolomic study. BMJ Open Respir. Res. 4, e000183. 10.1136/bmjresp-2017-000183 PMC5531310 28883924

