# Supplementary material for: Changes in serum metabolomics in idiopathic pulmonary fibrosis and effect of approved antifibrotic medication
Source: Front Pharmacol. 2022 Aug 17;13:837680. doi: 10.3389/fphar.2022.837680 (PMC9428132; doi:10.3389/fphar.2022.837680)
Supplement: Supplementary file 1 [file DataSheet1.docx]

Supplementary Material

Changes in Serum Metabolomics in Idiopathic Pulmonary Fibrosis and effect of approved antifibrotic medication

Benjamin Seeliger, Alfonso Carleo, Pedro David Wendel-Garcia, Jan Fuge, Ana Montes Worboys, Sven Schuchardt, Maria Molina-Molina, Antje Prasse

# Supplementary Methods

Biocrates MxP Quant 500 kit

This commercial kit measures 630 metabolites covering 14 small molecule and 12 different lipid classes using flow injection analysis tandem mass spectrometry (FIA-MS/MS) and liquid chromatography tandem mass spectrometry (LC-MS/MS) as previously described. Quantification of measurements was carried out using SCIEX Analyst software and was then imported in to the Biocrates MetIDQ software for analyte identification and calculation of concentrations.

# Supplementary Figures and Tables

## Supplementary Figures


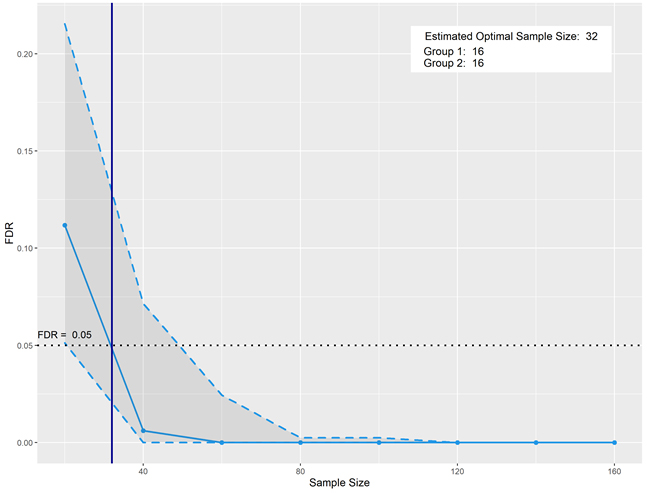


**Supplementary Figure S1** Group Size estimation using MetSizeR with an estimated significant rate of 15%, FDR 0.05, minimal group size of n=10 and a targeted sample size of 375.


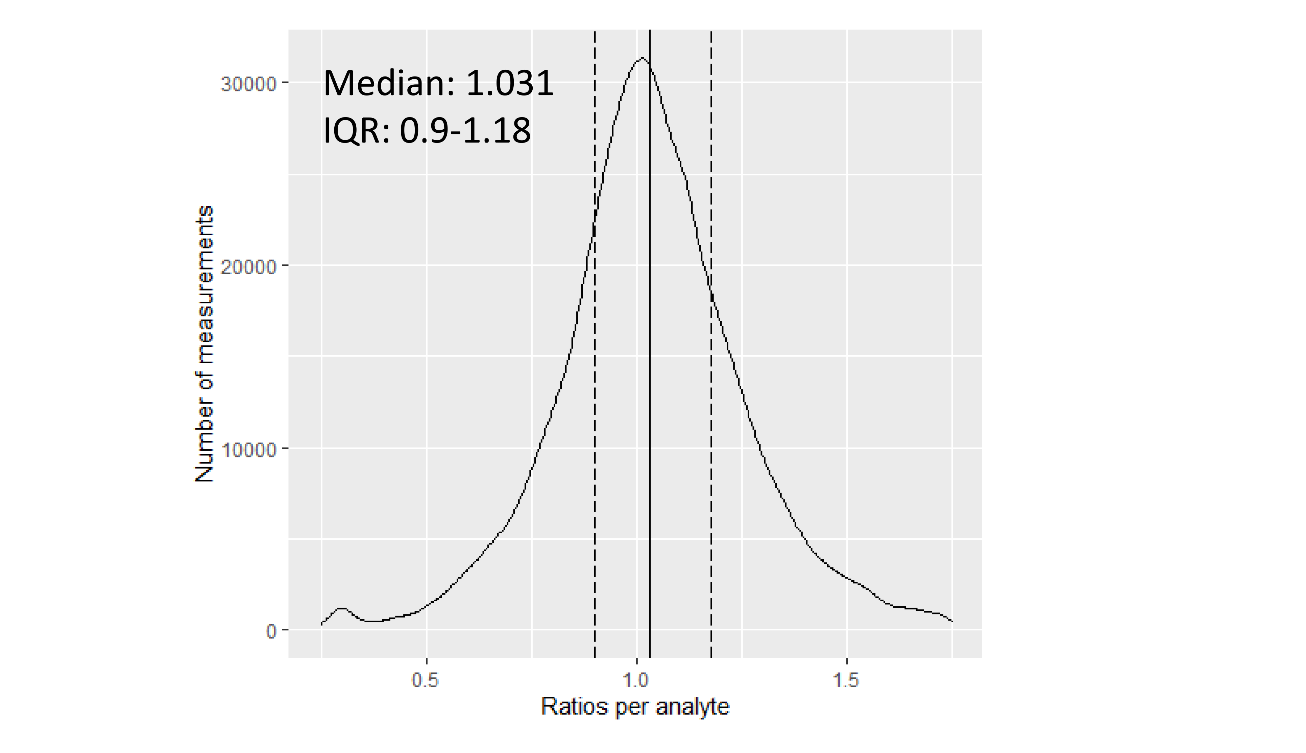


**Supplementary Figure S2** Ratios between each analyte of the original assay of cohort 1 and repeated measure of the same sample in another batch plotted as histogram.
